# Supplementary material for: Coupling of HSP72 α-Helix Subdomains by the Unexpected Irreversible Targeting of Lysine-56 over Cysteine-17; Coevolution of Covalent Bonding
Source: Molecules. 2020 Sep 16;25(18):4239. doi: 10.3390/molecules25184239 (PMC7570744; doi:10.3390/molecules25184239)
Supplement: Supplementary file 1 [file molecules-25-04239-s001.pdf]

Coupling of HSP72 α-helix subdomains by the unexpected irreversible targeting of Lysine-56 over Cysteine-17; coevolution of covalent bonding

Aimen Aljoundi[1], Ahmed El Rashedy[1], Patrick Appiah-Kubi[1], and Mahmoud E.S Soliman[1*]

[1]Molecular Bio-computation and Drug Design Laboratory

School of Health Sciences, University of KwaZulu-Natal, Westville Campus, Durban 4001, South Africa

*Corresponding Author: Mahmoud E.S. Soliman

Email: soliman@ukzn.ac.za

Telephone: +27 (0) 31 260 8048, Fax: +27 (0) 31 260 78

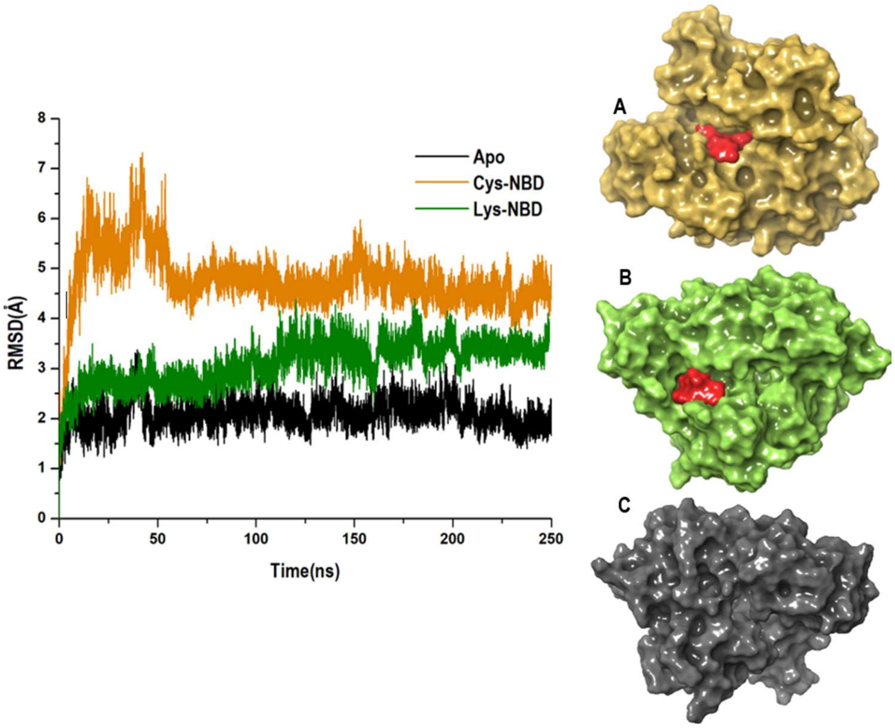

Figure S1. Comparative C-α RMSD plots showing the degree of stability and convergence of the studied systems over the 250ns MD simulation time; **[A]** Cysteine-NBD**[B]** Lysine-NBD **[C]** Apo.
